# Development of Adenoviral Delivery Systems to Target Hepatic Stellate Cells In Vivo

**DOI:** 10.1371/journal.pone.0067091

**Published:** 2013-06-18

**Authors:** Julia Reetz, Berit Genz, Claudia Meier, Bhavani S. Kowtharapu, Franziska Timm, Brigitte Vollmar, Ottmar Herchenröder, Kerstin Abshagen, Brigitte M. Pützer

**Affiliations:** 1 Institute of Experimental Gene Therapy and Cancer Research, Rostock University Medical School, Rostock, Germany; 2 Institute for Experimental Surgery, Rostock University Medical School, Rostock, Germany; Institute of Hepatology, Foundation for Liver Research, United Kingdom

## Abstract

Hepatic stellate cells (HSCs) are known as initiator cells that induce liver fibrosis upon intoxication or other noxes. Deactivation of this ongoing remodeling process of liver parenchyma into fibrotic tissue induced by HSCs is an interesting goal to be achieved by targeted genetic modification of HSCs. The most widely applied approach in gene therapy is the utilization of specifically targeted vectors based on Adenovirus (Ad) serotype 5. To narrow down the otherwise ubiquitous tropism of parental Ad, two modifications are required: a) ablating the native tropism and b) redirecting the vector particles towards a specific entity solely present on the cells of interest. Therefore, we designed a peptide of the nerve growth factor (NGF_p_) with specific affinity for the p75 neurotrophin receptor (p75NTR) present on HSCs. Coupling of this NGF_p_ to vector particles was done either via chemical conjugation using bifunctional polyethylene glycol (PEG) or, alternatively, by molecular bridging with a fusion protein specific for viral fiber knob and p75NTR. Both Ad vectors transmit the gene for the green fluorescent protein (GFP). GFP expression was monitored *in vitro* on primary murine HSCs as well as after systemic administration in mice with healthy and fibrotic livers using intravital fluorescence microscopy. Coupling of NGF_p_ to Ad via S11 and/or PEGylation resulted in markedly reduced liver tropism and an enhanced adenoviral-mediated gene transfer to HSCs. Transduction efficiency of both specific Ads was uniformly higher in fibrotic livers, whereas Ad.GFP-S11-NGF_p_ transduce activated HSCs better than Ad.GFP-PEG-NGF_p_. These experiments contribute to the development of a targeted gene transfer system to specifically deliver antifibrotic compounds into activated HSCs by systemically applied adenoviral vector modified with NGF_p_.

## Introduction

Due to the fundamental progress in elucidating the molecular mechanisms of human diseases, increasing numbers of therapeutic genes and cellular targets are available for gene therapy. In the meantime, the challenge is to develop gene delivery vectors that exhibit high target cell selectivity and efficiency *in vivo*.

Hepatic fibrosis represents a worldwide health problem with significant morbidity and mortality. Independent of its etiology, liver fibrosis mainly results from the activation of hepatic stellate cells (HSCs) which represent the major mesenchymal cell type in this organ [1]. Interestingly, the expression of p75 neurotrophin receptor (p75NTR) on activated HSCs is rapidly increased in liver fibrosis as well as in HSCs cultured *in vitro* [2]. Quiescent HSCs show low [3] and hepatocytes no expression of this receptor [4]. The currently available therapies and most of experimental drugs that influence HSC activity showed little efficiency *in vivo* [5]–[8]. In order to improve therapeutic efficiency, novel cell-specific delivery strategies towards HSCs are required.

The most commonly used vector system to transduce cells is based on adenovirus (Ad). Recent endeavors in the development of selective Ad vectors that target cells of interest and spare alteration of all other cells, have focused on modification of their broad natural tropism [9]. The present study describes methods to deliver therapeutic genes to HSCs *in vitro* and *in vivo* by targeted adenoviral gene transfer. Ad entry into host cells involves specific interactions between its fibers and cell surface receptors, above all the coxsackie- and adenovirus receptor (CAR) [10]. The virus' subsequent internalization requires another interaction of an arginine-glycine-aspartate (RGD) sequence on the Ad penton base with αVβ3 or αVβ5 integrins on the cell surface [11]. Ads infect dividing and non-dividing cells [12] and after administration, they do not integrate into the host genome [13]. This property makes them particularly attractive for gene therapeutic applications, where temporary gene expression is acceptable or even beneficial. The utilization of Ad as a targeted gene delivery system [14] is limited by its native tropism which allows the virus to infect a broad range of cells and tissues, in particular hepatocytes and Kupffer cells in the liver [15]. This property prevents selective gene transfer and imposes an increased risk of toxicity due to vector dissemination to non-targeted cells, even if Ad vectors are administered locally near or into the tissue of interest. This can be circumvented through cell-directed tropism-modification strategies [16]–[22] that reduce immune responses and increase safety and efficiency of systemically administrated Ad vectors.

Nerve growth factor (NGF) is a member of a family of structurally related proteins termed neurotrophins which bind to p75NTR [4]. The crystal structure of the extracellular domain of p75NTR complexed with its ligand NGF uncovered the amino acid stretch relevant for binding [23]. The latter study as well as the fact that activated HSCs induce p75NTR expression during fibrogenesis prompted us to design the peptide sequence designated NGF_p_ supposed to act as a targeting moiety. Here we applied two methods to modify the natural Ad vector tropism. The first method involved bridging vector and HSCs with a bispecific adapter molecule consisting of a single chain antibody fragment (S11) directed against the Ad fiber knob and NGF_p_ that recognizes p75NTR [7], [24], [25]. In the other approach, NGF_p_ was chemically conjugated to the Ad surface via polyethylene glycol, a procedure known as PEGylation [18], [22]. Both targeting approaches were performed on Ad vectors with unmodified natural tropism (Ad.GFP) that transmit the gene for the green fluorescent protein (GFP).

Our results demonstrate that both Ad retargeting strategies resulted in selective and enhanced adenoviral infection of HSCs, both *in vitro* and *in vivo*.

## Materials and Methods

### Isolation of primary liver cells

For the isolation of primary liver cells female Balb/c mice (Charles River Laboratories, Sulzfeld, Germany) were anesthetized and positioned supine on a warming pad. After laparotomy the inferior vena cava was cannulated with a 20 G needle, the portal vein was lanced, and the liver was perfused with different digestion solutions according to the protocol below. The isolation of HSCs was realized by an *in situ* pronase and collagenase perfusion followed by a density gradient centrifugation step as described elsewhere [26]. Briefly, livers were rinsed with an ethylene glycol tetra acidic (EGTA) solution (10 min, 10 ml/min) to wash all the blood out and then digested by perfusion with pronase E (0.6 mg/ml [2400 PU/ml], 5 min, 5 ml/min; Merck, Darmstadt, Germany) and collagenase D (0.32 mg/ml [0,071 U/ml], 6 min, 5 ml/min; Roche Diagnostics, Mannheim, Germany). The obtained cell suspension was filtered through a 100 µm mesh and washed two times with Geýs Balanced Salt Solution (GBSS), containing DNase I (2 mg/ml; Roche Diagnostics). The purification of HSCs from the other cells was transposed by density gradient centrifugation with 8.5% (w/v) Histodenz^TM^ (Sigma Aldrich Chemical Company, Steinheim, Germany) in GBSS. The pure cell pellet was resuspended in Dulbecco’s Modified Eagle Medium (DMEM) with low glucose and 10% FCS (PAA Laboratories, Pasching, Austria) and the cells were plated in cell culture dishes. Hepatocyte isolation was performed separately as described first by Seglen et al. [27]. Briefly, perfusion of the mouse liver with EGTA buffer for 10 min was followed by a digestion step with a collagenase 1A (0.05% [w/v]; Sigma Aldrich) solution for about 15 min, both at a flow rate of 15 ml/min. The acquired cell suspension was passed through a 100 µm cell mesh and hepatocytes were separated by centrifugation at 50 g (5 min, 4°C). The isolated cells were cultured on collagen A (1 mg/ml in PBS; Biochrom, Berlin, Germany) coated cell culture dishes with Williams E medium (PAN Biotech, Aidenbach, Germany) containing L-glutamine, 10% FCS and 100 nM dexamethasone (Sigma Aldrich).

### S11-NGF_p_ fusion protein cloning

For construction of the fusion protein S11-NGF_p_, the S11 cDNA from the pUC119-S11-Myc-His plasmid (a gift from Dr. R. Hawkins, Bristol University, UK) was subcloned into the pSecTag2C vector (Invitrogen, Groningen, NL) via *Not*I and *Sfi*I sites to obtain pSecTag2C+S11. For pSecTag2C+S11-NGF_p_, forward and reverse oligonucleotides for the coding sequence GACGCGGCCCAGCCGGCCGTGGAAAAAGGC AAAATTGATACCGCGA CCACCAAATGCGCGGCCCAGCCGGCCGGGGC were annealed, cleaved (*Sfi*I cleavage side underlined) and cloned into the *Sfi*I site of pSecTag2C+S11. After verifying the correct orientation via sequence analysis, lentiviral vector production was performed by transferring S11 or S11-NGF_p_ obtained from the corresponding pSecTag2C into the pWPXL vector including the Igκ leader sequence, His-, and *myc*-tag. After the cloning process we generated lentivirus particles in HEK293T cells and sequentially infected HEK293T cells to obtain a stable cell line which continuously secreted S11 or S11-NGF_p_, respectively.

### Purification of 6xHis-tag proteins

S11 or S11-NGF_p_ was purified from the cell culture media by nickel-affinity chromatography (Ni-NTA) (Qiagen Inc., Valencia, CA, USA). To do so, supernatant and Ni-NTA were mixed and incubated overnight (ON) at 4°C under rotation. The mixture was washed with buffer (20 mM sodium phosphate buffer, 0.5 M NaCl (Merck), pH 7.4) containing 20 mM imidazole (Invitrogen). Protein was eluted with 250 mM imidazole and dialyzed against phosphate buffered saline (PBS) at 4°C ON. Protein amounts were adjusted after Bradford assay. 5 or 10 µg were used for ELISA assays and 100 µg for Ad targeting assays *in vitro* and *in vivo*.

### Expression and purification of recombinant fiber

The knob domain of Ad serotype 5 with an N-terminal 6xHis-tag (kindly provided from Prof. Dr. H.J. Haisma, Groningen Research Institute of Pharmacy, Netherlands) was expressed in BL21 *E.coli* (Invitrogen). Bacteria were grown at 37°C and shaken 300 rpm in LB-media containing 100 µg/ml ampicillin and 25 µg/ml kanamycin until an OD_600_ of 0.8 – 1.0. Protein expression was induced with 2 mM isopropyl B-d-thiogalactoside (IPTG, Invitrogen). Bacteria were harvested by centrifugation at 4,000 g for 10 min. The pellet was resuspended in 2–5 volumes of sonication buffer (50 mM Na-phosphate (Fluka Analytical, St. Gallen, Switzerland), 300 mM NaCl 1 mg/ml lysozyme (Sigma); pH 7.8) and incubated for 30 min on ice. The suspension was sonicated 3 times for 10 s and centrifugated for 20 min at 10,000 g. The supernatant was added to Ni-NTA and treated as described above.

### Adenovirus vector production

Ad serotype 5-derived wild-type vector (Ad.GFP), expressing the green fluorescence protein (GFP), was generated by homologous recombination following cotransfection with pAdEasy1 in *E. coli* BJ5183. Ad vectors were propagated in HEK293 cells, purified by CsCl buoyant density centrifugation, and measured at OD_260_. *In vitro* adenoviral infections were carried out at multiplicities of infection (M.O.I) that allow a proper transduction of each cell line (M.O.I = 10 for hepatocytes, M.O.I = 5 for HSCs).

### Adenovirus vector modification

A way of Ad targeting is directing the vector towards individual cellular receptors. This was achieved by linking NGF_p_, specific for p75NTR binding, to viral surface proteins via chemical coupling or adapter molecules [7]. The peptide sequence used in this study (KTTATDIKGKEV) comprises the amino acids 25–36 of the full-length NGF protein [28]. The adapter molecules S11 or S11-NGF_p_ (100 µg each) were preincubated with Ad.GFP for 60 min at 37°C in serum-free DMEM before adding the mixture for 1 h to the cells. Under chemical coupling covalent linkage of the synthetic NGF_p_ to Ad.GFP by PEGylation was performed. Briefly, bifunctional PEG (Sunbright, NOF Corporation, Japan) was added to the virus with 5% w/v by stirring for 1 h at room temperature (RT) [29]. CsCl gradient centrifugation was performed to separate unbound PEG from the PEGylated virus followed by dialysis against PBS containing 5% sucrose ON at 4°C. Peptide dissolved in PBS was added to the PEGylated virus at a concentration of 1 mM while stirring for 4 h at 4°C. Separation of unreacted peptide was achieved by dialysis ON in PBS containing 5% sucrose.

### Adenoviral targeting assay

After isolation, primary mouse hepatocytes and HSCs were seeded in 6-well plates at a density of 1×10^5^ cells and grown at 37°C in a 5% CO_2_ humidified atmosphere. After 24 h in culture, cells were infected with Ad.GFP that carried S11 or S11-NGF_p_ and Ad.GFP-PEG-NGF_p_ for 45 min at 37°C. Before adding fresh media, cells were washed twice with PBS and maintained in culture (hepatocytes for 2 days and HSCs for 3 days). Gene transfer into the cells was measured by GFP quantification with a Laser Scanning Microscope (Carl Zeiss AG, Oberkochen, Germany). The infection of HSCs by adenoviruses was determined by counting GFP positive HSCs in relation to p75NTR stained cells.

### Immunoblotting

Primary mouse hepatocytes and HSCs were lysed in lysisbuffer (50 mM Tris-Cl, 150 mM NaCl, 1% NP-40, 0.5% sodium deoxycholate, 0.1% SDS, 10x protease inhibitor). Equal amounts of cellular protein (10 µg) were separated by sodium dodecyl sulfate polyacrylamide gel electrophoresis (SDS-PAGE), transferred to nitrocellulose membranes (Amersham Biosciences, Freiburg, Germany), and probed with appropriate primary antibodies: anti-CAR, anti-p75NTR, anti-Integrin-αν5, anti-Integrin-β3, anti-Integrin-β5 (all 1:1,000; Santa Cruz Biotechnology, Santa Cruz, CA, USA), anti-*myc*-tag (1:100; Invitrogen), and anti-β-actin (1:20,000; Sigma) for 4°C ON. The corresponding HRP-conjugated secondary antibodies incubated for 1 h at RT were detected using ECL Western blot reagents (Amersham Biosciences).

### Immunofluorescence and laser scanning microscopy

Cells were seeded on glass cover slips and fixed with 10% formaldehyde/PBS after 24 h. After fixation, slides were washed and blocked with PBS/1% BSA for 1 h. Anti-p75NTR antibody or anti-*myc-*tag antibody were added ON at 4°C. After washing with PBS the slides were incubated with appropriate secondary AlexaFluor^594^ antibody (Life Technologies GmbH, Darmstadt, Germany) and subjected to fluorescence-activated laser scanning microscopy (Zeiss).

### ELISA

The S11 protein binding capacity to the adenoviral fiber was analyzed by an enzyme-linked immunosorbent assay (ELISA). A Ni-NTA HisSorb 96-well plate (Qiagen) was incubated with 1 µg of recombinant Ad5 fiber proteins. The S11 and S11-NGF_p_ were added to the wells and incubated for 2 h at RT. ELISA was performed using the primary anti-*myc-*tag antibody and the corresponding secondary HRP conjugated antibody and followed by addition of a chromogen substrate, 3,3′,5,5′-tetramethylbenzidine (TMB, Promega GmbH, Mannheim, Germany). After washing the cells, binding of the fusion proteins was determined by measuring the absorbance at 490 nm (3 independent experiments with n = 8 per condition).

### NGF_p_ and p75NTR binding assays

For the binding affinity assay primary isolated mouse HSCs were seeded onto Maxisorp plates (NUNC, Roskilde, Denmark). After 24 h in culture, cells were maintained 3 h in 0.5% FCS containing media, washed with PBS, and S11 or S11-NGF_p_ was added in a concentration dependent manner (5 µg and 10 µg). After 3 h incubation at 37°C cells were washed 3 times with PBS. ELISA was performed using anti-*myc-*tag antibody and the corresponding HRP-conjugated secondary antibody. For the competition assay, primary isolated mouse HSCs were seeded as described for the binding assay. After washing, cells were incubated with 8 µg of anti-p75NTR or unspecific IgG antibody for 3 h before adding 50 µg of S11-NGF_p_ in 0.5% FCS containing media at 37°C ON. To quantify the binding rates after the competition assay, anti-*myc-*tag antibody and the corresponding HRP-conjugated secondary antibody was used to perform the ELISA (3 independent experiments with n = 8 per condition).

### Animals and infection experiments

Male Balb/c mice (Charles River Laboratories) at an age of 10 to 12 weeks were kept on standard pellet food and water *ad libitum* with a 12 h day-and-night-cycle. Animals were anesthetized by an intraperitoneal injection of ketamine (90 mg/kg bw) and xylazine (25 mg/kg bw) and placed on a warming pad to maintain the body temperature at 37°C. The jugular vein was exposed and the virus suspension (1×10^11^ particles in PBS with a final volume of 200 µl) was injected slowly using an insulin needle (0.3×12 mm, B. Braun Melsungen AG, Melsungen, Germany) (n = 4 animals per vector system). After 48 h, intravital fluorescence microscopic documentation of GFP- and vitamin A-fluorescence in liver cells was performed. After blood sampling for liver enzyme analysis, liver, lungs, and brain were fixed in 4% saline-buffered formalin or frozen at −80°C for histological and mRNA analysis. All experiments were approved by the local government (LALLF M-V/TSD/7221.3-1.2-049/09) and performed in accordance with the German legislation on protection of animals and the National Institutes of *“Health Guide for the Care and Use of Laboratory Animals”* (Institute of Laboratory Animal Resources, National Research Council; NIH publication 86-23 revised 1985).

### Animal model of secondary biliary fibrosis (BDL)

Under inhalation anesthesia with isoflurane male Balb/c mice were placed on a heat pad and laparatomised. The common bile duct was isolated, ligated three times with non-resorbable suture (polyester 5–0; Catgut, Markneukirchen, Germany) and cut between the two gut-near ligatures [30]. The abdominal muscle and skin layer were stitched and mice were treated with metamizole as analgesic in their drinking water. The virus injection was performed on day 7 after BDL (n = 4 animals per vector system).

### Intravital fluorescence microscopy and off-line quantitative analysis of HSC-infection

Virus treated and untreated control mice were anesthetized and placed on a 37°C warming pad. After longitudinal and transversal abdominal section, the left lateral liver lobe was mobilized and placed in a horizontal position on a fixed plasticine stage to avoid respiratory movement and facilitate a planar surface to allow an ideal focus for microscopy. The exposed lobe was moistened with sodium chloride and covered with a glass slide to protect it from drying. After termination of the microscopic recordings, blood was collected from the vena cava inferior and the animals were delivered by exsanguination. The intravital microscopic studies were realized by using a modified fluorescence microscope (Axiotech, Zeiss) equipped with a 100 watt mercury lamp and filter stacks for blue (excitation/emission wavelength: 450–490 nm/>520 nm) and ultraviolet epi-illumination (UV, 330–390 nm/>430 nm). Blue epi-illumination was used to detect the green fluorescence of GFP expressing cells and UV epi-illumination was used to visualize the autofluorescence of vitamin A in the lipid droplets of HSCs. The observation of the liver cells was carried out with 10x (EC Plan-NEOFLUAR, 10x/0.3, Zeiss), 20x (LUCPlanFLN, 20x/0.45, Olympus, Hamburg, Germany), and 63x (ACHROPLAN, 63x/0.95w, Zeiss) objectives and recorded by a video camera (FK-CM-2412-201-IQ-R4, Pieper, Berlin, Germany). Microscopic images were transferred to and saved by a digital video system (DVD, PMR-ES35, Panasonic, Kadoma, Japan). Afterwards, they were analyzed off-line using a computer-assisted image analysis system (CapImage; Zeintl, Heidelberg, Germany). HSCs were identified as intense areas of vitamin A autofluorescence, which rapidly faded upon UV epi-illumination. GFP expressing cells were identified as HSCs by both green and ultraviolet fluorescence and their identical intrahepatic localization.

Quantitative analysis of infected HSCs was performed off-line in a 388-fold magnification using the computer-assisted image analysis system (CapImage) as described previously [31], [32]. Five to six microscopic fields per mouse were randomly selected for two-dimensional data analysis. For the assessment of GFP-fluorescence, grey levels were determined densitometrically with subsequent automatic calculation of the area of positive signals as percent of the whole area of observation. Distribution of HSC-associated vitamin A autofluorescence was assessed by densitometric recording of positive sites of fluorescence within the same frame. Due to the photobleaching property of vitamin A autofluorescence, only images acquired in the first second of UV epi-illumination were used for data analysis. The area of positive vitamin A sites was calculated automatically as percent of the whole area of the single frame. Finally, GFP positive HSCs were given in percent of the total vitamin A autofluorescence-associated sites per observation area.

### Immunohistochemistry

Liver tissue of animals was formalin-fixed,embedded in paraffin, and cut into 5 µm sections. To analyze the infection by the administered vectors, the slides were immunostained ON at 4°C for GFP (1:1,000; Abcam, Cambridge, UK) and p75NTR (1:1,000; Abcam). The secondary antibodies goat-anti-rabbit-AP (alkaline phosphatase; Dako Cytomation, Hamburg, Germany) and goat-anti-rabbit-HRP (horseradish peroxidase; Dako) were incubated for 1 h at RT. The signals were then detected by using liquid permanent red for GFP (Dako) or 3, 3′-diaminobenzidine for p75NTR (DAB, Dako). Additionally, all tissue slides were counterstained with hematoxylin.

### RT-PCR

Frozen tissue was lysed in RLT buffer and total RNA was isolated by using RNeasy RNA isolation kit (Qiagen). Quantity and quality of the isolated RNA was evaluated by nanodrop spectrophotometer technology (Thermo Scientific, Wilmington, DE, USA). Then 2 µg of total RNA was reverse transcribed into first strand cDNA using oligo(dT)18 primer (Biolabs, Frankfurt am Main, Germany) and Superscript II RNaseH-Reverse Transcriptase (Invitrogen, Karlsruhe, Germany). Primers used for amplification are: GFP forward: 5′-ATCTTCTTCAAGGACGACGG-3′, reverse: 5′-CTGTTGTAGTTGTACTCCAGC-3′; GAPDH forward: 5′-CGTCCCGTAGACAAAATGTG-3′, reverse: 5-GAATTTGCCGTGAGTGGAGT-3′.

### Immunofluorescence double-staining

To identify infected cells by co-expression of GFP and the HSC markers αSMA (activated HSC) or GFAP (quiescent and activated HSC), slides were immunostained for GFP (1:100; GeneTex, Irvine, USA) and αSMA (1:600; Abcam) or GFAP (1:1000; Abcam). The secondary antibodies goat-anti-rabbit-DyLight^488^ (Abcam) and donkey-anti-goat-DyLight^594^ (Dianova, Hamburg, Germany) were incubated for 1 h at RT. Additionally, all tissue slides were counterstained with DAPI (1:1000; AppliChem, Darmstadt, Germany) 10 min at RT and with sudan black (Santa Cruz) 15 min at RT. The signals were then detected by using a fluorescence microscope (Leica, Wetzlar, Germany).

### Analysis of ALT and AST activities

Plasma activities of alanine aminotransferase (ALT) and aspartate aminotransferase (AST) were measured spectrophotometrically as indicators of hepatocellular disintegration.

### Neurite outgrowth assay

PC12 cells (CLS Cell Lines Service, Eppelheim, Germany) were cultured at 37°C under 5% CO_2_ in RPMI medium supplemented with 15% heat-inactivated horse serum (HS), 1% penicillin, and 1% streptomycin. Cells were plated onto 24-well tissue culture plates (8×103 cells/well) coated with collagen. To differentiate PC12 cells, 24 hours after plating medium was replaced with RPMI containing 0.5% HS, 1% penicillin, and 1% streptomycin with NGF (50 ng/ml), NGF_p_ (50 ng/ml), S11-NGF_p_ (50 ng/ml) or S11-NGF_p_ bound to Ad.GFP (100 µg S11-NGF_p_ bound to Ad.GFP MOI 5). NGF-induced cell differentiation is defined by the presence of at least one outgrown neurite equivalent to the length of the cell body diameter. Neurite outgrowth was visualized by phase contrast microscopy (Zeiss).

### Statistical analysis

Results are presented as the mean of three-four independent experiments ± standard deviation. Differences in *in vitro* experiments were analyzed using a two-tailed paired Student´s *t*-test (**p<0.02). For quantitative data of the *in vivo* experiments, significance of differences between the groups was tested by One way ANOVA on Ranks (Kruskal-Wallis) (*p<0.05).

## Results

### Two approaches to specifically target HSCs

The present study aimed at specifically directing Ad vectors to HCSs while sparing hepatocytes and other liver cells. Under *in vitro* conditions hepatocytes and HSCs can be transduced with unmodified Ad vectors, where hepatocytes are transduced more efficient at lower M.O.I. than HCSs (data not shown). Vector entry into both cell types takes place after attachment of the trimeric fiber protein to the rather abundant CAR expressed on the cell plasma membrane, and subsequently secondary CAR-independent interactions between the penton base and the cell surface take place. Blocking the essential initial binding step either by removing the CAR-binding domain from the viral knob or by other ways of blocking this moiety completely prevents the Ad vector's ability to attach to and thereafter, to transduce any cells present in or derived from the liver.

Here we applied and compared two different principles to transduce HSCs and concomitantly, leave other liver cells unaffected. The system is based on the affinity of a short peptide termed NGF_p_, previously shown to exhibit specific binding affinity for p75NTR [28] present on HSCs but not on other liver cells [2]. Ad.GFP vectors were equipped with NGF_p_ either as part of a chimeric single-chain antibody fragment termed S11 [33] or by coating the vector particles with synthetic NGF_p_ by means of the bifunctional cross-linker PEG [29]. The functionality of both approaches is demonstrated and advantages and disadvantages are discussed below.

### Construction, production and functionality of the single chain antibody S11 and its derivative S11-NGF_p_

A single-chain antibody with high affinity for the CAR-binding portion on the adenoviral fiber knob was utilized to ablate the native binding moiety of viral particles. This S11 antibody which is artificially equipped with both, a 6xHis-tag component for purification and a *myc*-tag-element for detection purposes, binds to the Ad fiber knob and prevents the latter from interaction with CAR and, thus, ablates the vector's native tropism. This construct was further modified by genetically integrating the coding sequence for NGF_p_. The derivative S11-NGF_p_ maintains the ability to block adenoviral interaction with CAR and enables binding to the alternative receptor p75NTR on HSCs.

**Figure 1:**
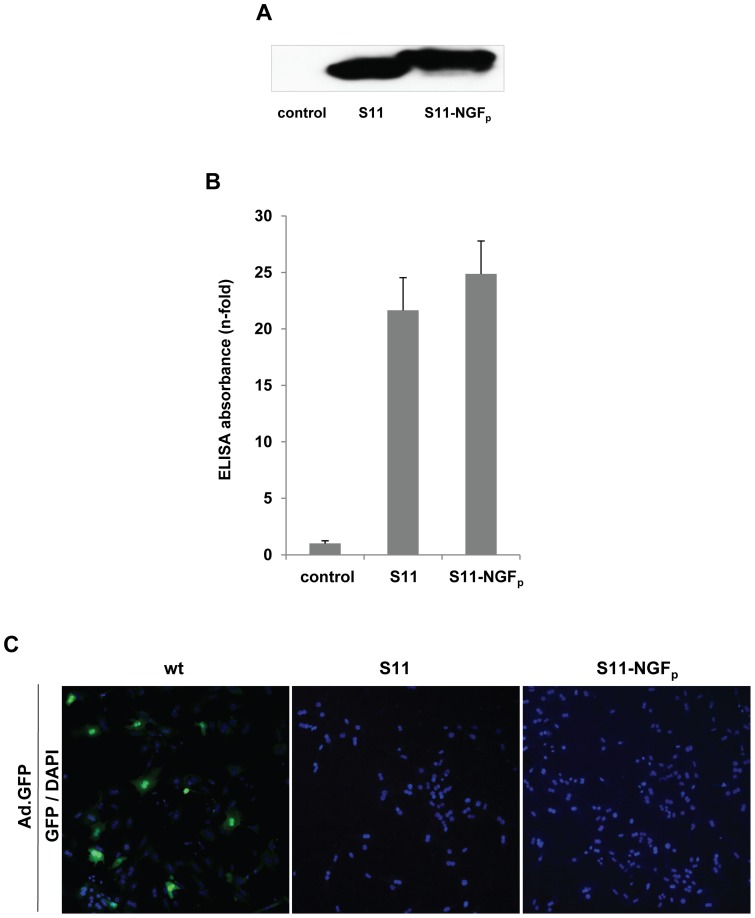
Construction, production and functionality of the single chain antibody S11 and its derivative S11-NGF_p_. (A) Western blot analysis of Ni-NTA-purified S11 and S11-NGF_p_ protein produced in HEK293T cells using anti-*myc*-tag antibody (MW of approximately 40 kDa). (B) Purified S11 and S11-NGF_p_ was analyzed for binding to recombinant Ad fiber knob using ELISA assay. S11 served as a positive control and control comprises buffer only. (C) Transduction of hepatocytes with Ad.GFP incubated with S11 and S11-NGF_p_ results in ablation of native Ad tropism. Ad.GFP (wt) served as an infection efficiency control.

To evaluate protein expression and purification yield, Western blot analysis was performed (Fig. 1A). To further analyze the effect of the NGF_p_ when combined with S11, a recombinant Ad5 fiber ELISA assay was established. The ELISA plates were coated with purified Ad fibers and subsequently incubated with S11 or S11-NGF_p_. As shown in Fig. 1B, the high binding efficiency of S11 and S11-NGF_p_ to the fiber demonstrates the functionality of the S11 part in both constructs compared to the control.

To further demonstrate that the S11 portions of both constructs bind to the vector and ablate CAR binding and virus uptake similarly, primary hepatocytes were infected with Ad.GFP previously incubated with S11 or S11-NGF_p_, respectively. Adenoviral transgene expression was monitored by fluorescence microscopy. The transduction efficiency of the cells was completely ablated by incubating the virus with either of the proteins prior to infection, whereas unmodified Ad.GFP induced transgene expression (Fig. 1C). These data confirmed the feasibility of the S11 constructs [25] and demonstrated identical characteristics of its derivative S11-NGF_p_ to ablate viral entry into primary hepatocytes.

### NGF_p_ binds specifically to p75NTR

**Figure 2:**
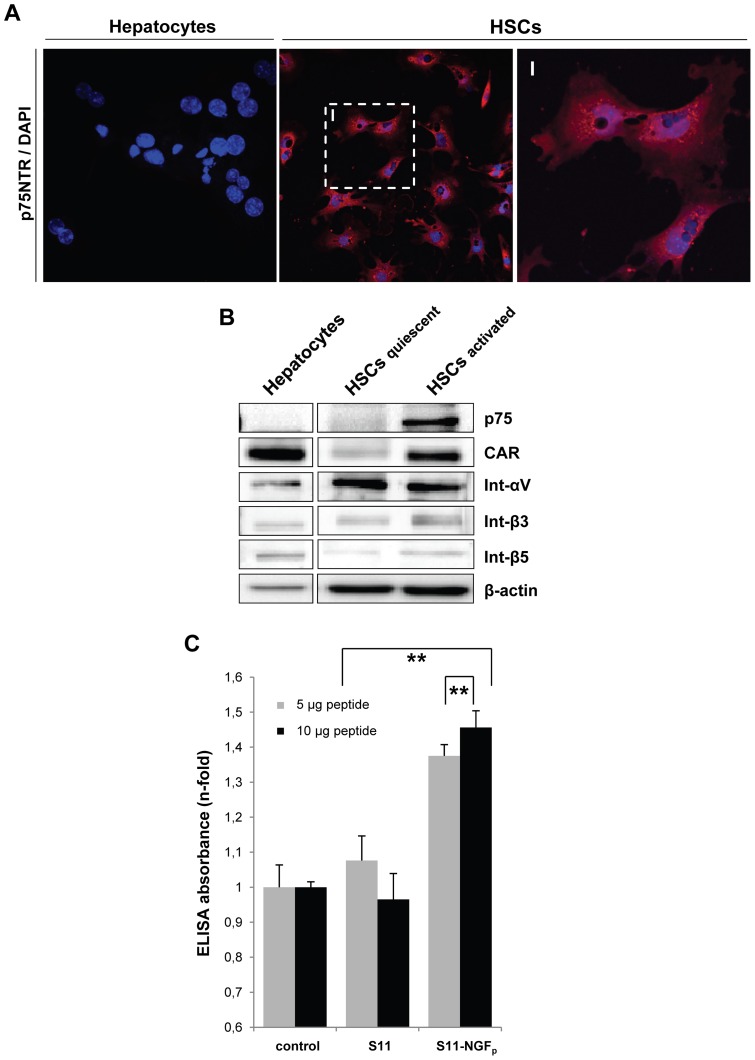
Analysis of cell surface receptor expression on hepatocytes or HSCs and NGFp binding specificity to p75NTR. (A) Fluorescence microscopic images of p75NTR expression (red) on primary hepatocytes and HSCs (DAPI stained nuclei are blue) showing a HSC-specific expression of p75NTR (10x). (B) Cell lysates of freshly isolated hepatocytes, quiescent and culture-activated HSCs were analyzed for expression of p75NTR, CAR, αV, β3 and β5 integrins (Int). (C, D) Binding of S11 and S11-NGF_p_ to p75NTR positive cells (HSCs) was evaluated by ELISA and immunofluorescence using anti-*myc*-tag antibody (scale bar, 20 μm). (E) ELISA competition assay for analysis of specific NGF_p_/p75NTR binding revealed that preincubation with anti-p75NTR antibody decreased S11-NGF_p_ binding to p75NTR on HSCs to the same level as control IgG alone (**p<0.02). Magnified inserts, in (A, D) are labeled with roman numerals.

To achieve efficient gene transfer to HSCs, the utilization of a targeted adenovirus as a gene delivery system is needed. Therefore, we analyzed p75NTR expression on primary isolated liver cells – hepatocytes and HSCs – using immunofluorescence microscopy (Fig. 2A) and Western Blot analysis (Fig. 2B). As already described in literature [2], our data confirmed that HSCs express p75NTR depending on their activation level, while hepatocytes do not. Fresh isolated HSCs, i.e. quiescent HSCs, expressed very low levels of p75NTR which was upregulated during the activation process (Fig. 2B). CAR was expressed analogous. In contrast, we figured out that all three integrins (αV, β3 and β5) which were studied because of their role as co-receptors in virus internalization were expressed in hepatocytes as well as in quiescent and activated HSCs. Because of these findings we estimated p75NTR on HSCs as a valid candidate for vector targeting approaches.

The next step was to verify the ability of NGF_p,_ the candidate cell-binding component of S11-NGF_p_, to bind p75NTR by a cell ELISA assay. Therefore, HSCs were seeded in 96-well plates and S11 or S11-NGF_p_ was added at different concentrations (5 or 10 µg). A significant and concentration-dependent binding of S11-NGF_p_ to the cells is shown in Fig. 2C. To underline these data, immunostaining of HSCs incubated with S11 or S11-NGF_p_ was performed using an anti-*myc-*tag antibody. Positive staining in the S11-NGF_p_ approach (Fig. 2D) indicates a specific NGF_p_-p75NTR interaction, while S11 and control group did not yield a positive signal. These results strengthened the supposition that entry of Ad.GFP studded with S11-NGF_p_ is dependent on p75NTR which is exclusively present on HSCs and alternatively, provide evidence that S11 alone has no unspecific binding effect on cultured HSCs.

Additionally, the interaction between NGF_p_ and p75NTR on HSCs was confirmed using a monoclonal anti-p75NTR antibody as a direct competitor of NGF_p_ to the same binding side of p75NTR. Thus, cells were preincubated with anti-p75NTR 3 h before adding S11-NGF_p_ to prevent binding of the fusion protein to the receptor. Preincubation with anti-p75NTR decreased S11-NGF_p_ binding to p75NTR on HSCs to the same level as control IgG alone and confirmed competition of both for the identical epitope (Fig. 2E). These data validate the binding specificity of NGF_p_ to p75NTR present on HSCs. On the background of these observations, we attempted to target Ad vectors utilizing S11-NGF_p_ to specifically yield transgene expression in HSCs and spare hepatocyte infection.

### Selective infection of HSCs by targeted Ad vector

**Figure 3:**
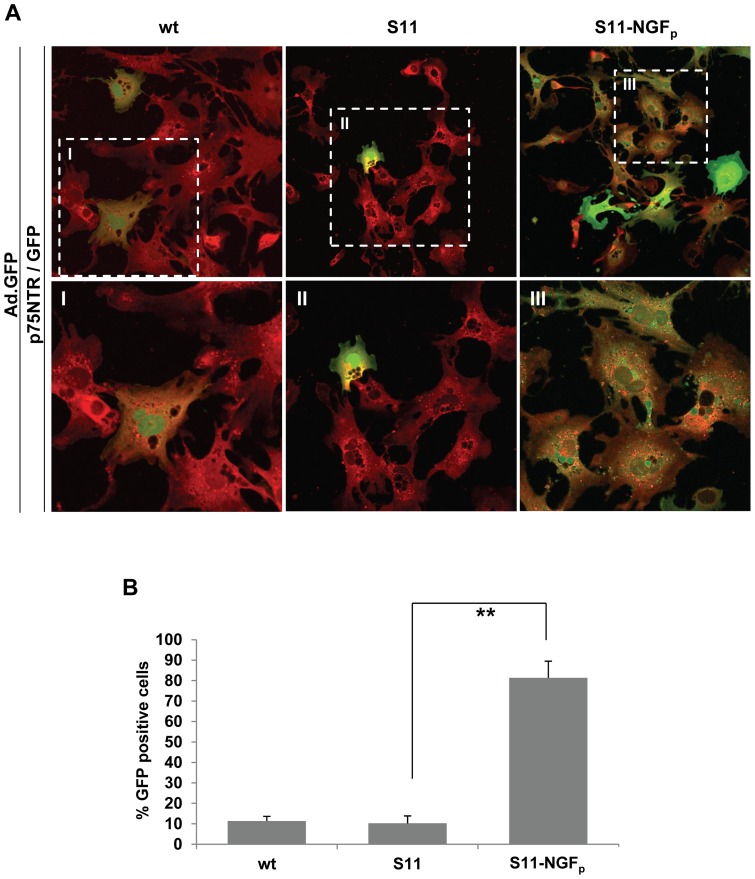
Selective infection of HSCs by S11-NGF_p_ targeted Ad vector. (A) After exposure of HSCs to Ad.GFP-S11-NGF_p_ a higher transduction rate was detected by GFP expression (green) using fluorescence microscopy compared to wild-type (wt) and S11 bound Ad.GFP. Simultaneous staining of p75NTR (red) on HSCs indicates NGF_p_ based cell infection (40x). (B) Quantification of GFP positive cells revealed an infection efficiency of approximately 80% using Ad.GFP-S11-NGF_p_ (**p<0.02). Magnified inserts are labeled as indicated (I–III).

For this rather elegant approach using S11-NGF_p_ to direct the vector exclusively to HSCs via their p75NTR, protein production was performed in eukaryotic cells. S11-NGF_p_ was purified and concentrated by nickel-affinity chromatography and high yield of pure protein without loss of activity for *in vitro* studies was ensured. To determine targeted adenoviral infection mediated by S11-NGF_p_, the fusion protein was titrated in an Ad infection experiment on HSCs, using Ad.GFP with a M.O.I of 5 (data not shown). For all further experiments, the optimal ratio of fusion protein per virus particle was determined as 100 µg of S11 or S11-NGF_p_ respectively, with Ad.GFP at a M.O.I of 5. The efficiency of NGF_p_ mediated Ad.GFP vector targeting was assessed by transgene expression. Cells were infected with Ad.GFP previously incubated with the optimal amounts of S11 or S11-NGF_p_. Three days after infection, as Ad.GFP alone or the vector incubated with S11 showed no significant transduction, Ad.GFP-S11-NGF_p_ strongly transduced HSCs (Fig. 3). Quantitative analysis of GFP positive HSCs revealed an infection efficiency of 80% using Ad.GFP-S11-NGF_p_, whereas only 10% of HSCs were infected by wild-type virus and S11 (Fig. 3B). Together with the data presented in Fig. 2, these results confirm an efficient and specific viral entry into HSCs at low M.O.I which is mediated by NGF_p_-p75NTR binding.

### Ad vector PEGylation, infection and efficiency

Our study describes a new strategy for retargeting adenoviral vectors to HSCs ablating the native viral tropism and simultaneously redirecting the vector to a specific entity on the desired target cells. Towards this goal, we successfully combined a strategy to ablate native adenoviral cell binding with an adapter derived from a single chain antibody fragment that bridges the Ad fiber and cell surface receptor p75NTR. In comparison, covalent modification of Ad vector capsid with PEG has been proven to aid in overcoming hurdles like Ad gene delivery to unspecific tissues or cells *in vivo*. In previous reports we demonstrated selective gene delivery via PEGylated Ad vectors to hippocampal stem cells [21] and neoplastic tissue [22] *in vivo*.

**Figure 4:**
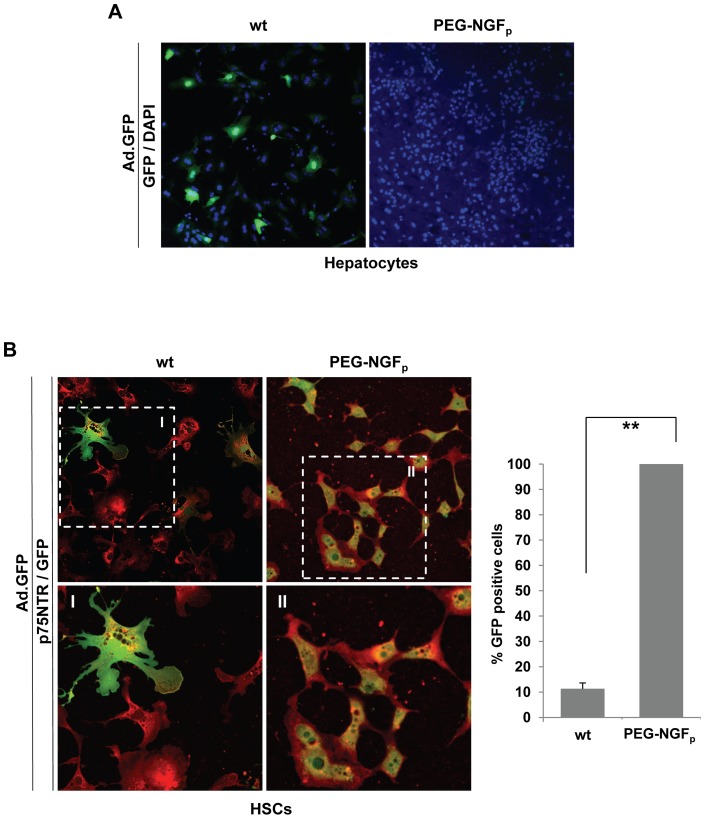
Ad vector PEGylation, infection and transduction efficiency. (A) In comparison to wild-type Ad.GFP (wt), coupling of NGF_p_ to Ad.GFP by PEGylation ablates hepatocellular infection, whereas (B) HSCs show GFP expression (green) when infected with Ad.GFP-PEG-NGF_p_. Positivity of HSCs for p75NTR (red) underlines NGF_p_ driven cell infection (40x). Quantification of GFP positive cells revealed a 100% infection efficiency using Ad.GFP-PEG-NGF_p_ (B, right panel) (**p<0.02). Magnified inserts are labeled with roman numerals.

In the present study, we compared the bispecific adapter approach with the PEGylation system, where NGF_p_ is covalently linked to the adenoviral surface. The infectivity of such vector particles was explored *in vitro* on primary hepatocytes (Fig. 4A) and HSCs (Fig. 4B) by analyzing GFP transgene expression. The data show that Ad.GFP PEGylated with NGF_p_ specifically transduced all HSCs (Fig. 4B, right), while hepatocytes showed no transgene expression due to the PEG-driven virus ablation and absence of p75NTR (Fig. 4A). Taken together, PEGylated Ad.GFP exhibiting NGF_p_ at its surface allows highly efficient gene transfer restricted to p75NTR positive cells.

The next step was to prove our targeting systems *in vivo* to achieve specific gene delivery into HSCs, concurrently avoiding its binding to hepatocytes.

### Intravital fluorescence microscopic analysis of infected mice livers

**Figure 5:**
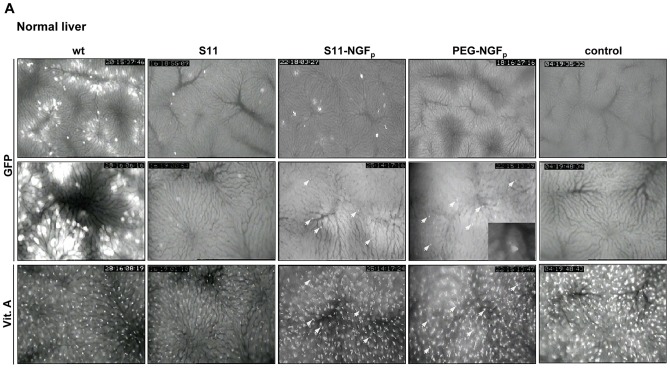
*In vivo* microscopic analysis of infected mice livers. Representative intravital fluorescence microscopic images of (A) normal and (B) fibrotic liver tissue, of non-infected mice livers (control) and mice livers infected with the wild-type Ad.GFP (wt) as well as different modifications of Ad.GFP: binding of bispecific adapter molecule S11 alone (S11); S11 coupled with NGF_p_ (S11-NGF_p_) and PEGylated Ad.GFP coupled with NGF_p_ (PEG-NGF_p_). Images in the upper (10x) and mid (20x) panel display green fluorescence of GFP expressing cells by using blue epi-illumination. UV epi-illumination was used to visualize the autofluorescence of vitamin A in the lipid droplets of HSCs (lower panel, 20x). GFP expressing cells were identified as HSCs by comparison of both green and ultraviolet fluorescence and their identical intrahepatic localization (A, arrows). Representative images of GFP positive quiescent and activated HSCs of normal (A) and fibrotic (B) livers are displayed in higher magnification (63x objective) as inserts or (C) with corresponding vitamin A autofluorescence demonstrating identical localization of both fluorescence signals. (D, left) Quantitative analysis of GFP signals in fibrotic mice. Screen displays of the CapImage software showing the densitometric recording of positive sites of GFP fluorescence (upper panel) and vitamin A (lower panel) autofluorescences per frame representative for S11-NGF_p_. Quantification of GFP positive area given in percent of the total vitamin A autofluorescence-associated area per observation field (right). Untreated animals served as control (*p<0.05). (E) Co-expression of GFP and GFAP, a marker for both quiescent and activated HSCs, detected by immunofluorescence. (F) Co-expression of GFP and HSC specific marker αSMA, detected by immunofluorescence.

To analyze whether the *in vivo* tropism of virus particles was altered after modification and coupling of NGF_p_, GFP expression of mouse livers was visualized 48 h after i.v. injection of the differentially modified virus particles using intravital fluorescence microscopy. As expected, *in vivo* analysis of livers infected with wild-type virus revealed GFP expression in numerous hepatocytes, primarily in periportal and midzonal areas of the liver lobules (Fig. 5A). Adenoviral modification by S11 reduced hepatocellular uptake extensively, only a few hepatocytes showed GFP expression. By targeting of Ad.GFP-S11 through coupling of NGF_p_, several transduced HSCs were observed as indicated by GFP colocalization with vitamin A.

When injected i.v. into mice, PEGylated wild-type virus did not show hepatocyte-tropism as evidenced by the complete absence of hepatocellular GFP fluorescence (data not shown). In accordance to this, GFP expression in mice receiving Ad.GFP-PEG-NGF_p_ appeared to be restricted to nonparenchymal cells, as shown in Fig. 5A by a spotty appearance of GFP fluorescence signals near the sinusoidal vessels. Nearly all of these spots were identified as HSCs due to their colocalization with vitamin A.

Expression of p75NTR is increased in activated HSCs during fibrogenesis. To address a p75NTR targeted adenovirus and obtain a high transduction efficiency, we tested both adenoviral targeting systems in BDL mice (Fig. 5B) expecting higher transduction rate. Results show that the global transduction rate of the wild-type Ad in the injured livers was less than in normal livers. Interestingly, in fibrotic livers nonparenchymal cells were transduced with greater frequency with wild-type Ad than hepatocytes. Furthermore, in injured livers infected with the NGF_p_-coupled viruses, a markedly increased GFP expression in cells located next to sinusoids was observed. Higher magnification of infected cells (Fig. 5C) clearly showed spotty GFP signals of quiescent HSCs in normal livers, whereas in fibrotic livers GFP signals had the form of activated HSCs. All GFP signals were colocated with vitamin A autofluorescence. Quantification of vitamin A autofluorescence area revealed that 8–9% of the whole liver area emits HSC-associated vitamin A autofluorescence (data not shown). This is in accordance to the number of HSCs within the liver, representing about 5–8% of all liver cells. The rate of GFP positive HSCs was significantly increased in fibrotic livers infected with S11-NGF_p_ when compared to control or S11 infected animals (Fig. 5D). In mice infected with S11-NGF_p_ 30% of the total vitamin A positive HSCs were also positive for GFP. Usage of PEG-NGF_p_ resulted in an HSC-infection rate of 20%. That infected cells are indeed quiescent and activated HSCs was evidenced by immunofluorescence double-labeling of GFP and HSC markers GFAP (Fig. 5E) and αSMA (Fig. 5F) demonstrating their colocalization. Taking together, these data suggest that both targeting systems effectively reduced liver tropism and increased transgene expression in quiescent and particularly in activated HSCs.

### Immunohistochemical analysis of GFP positive cells

**Figure 6:**
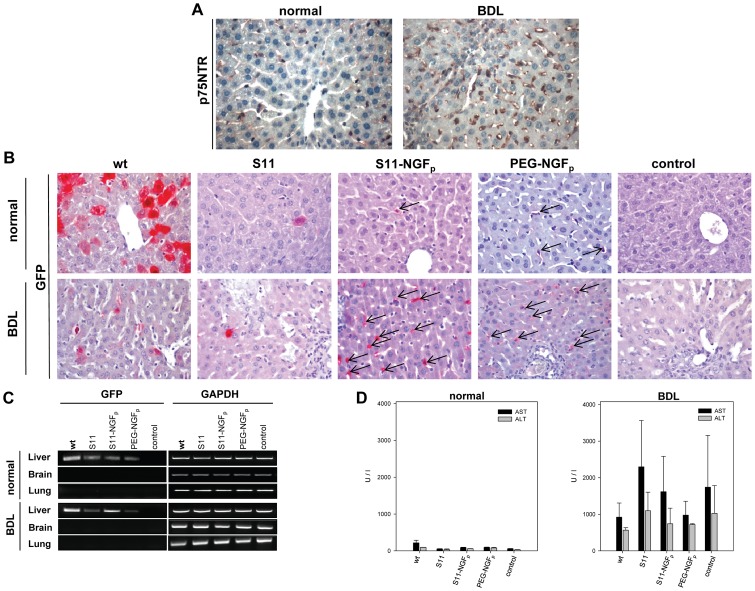
Immunohistochemical analysis of infected mice livers and vector biodistribution. (A) Representative immunohistochemical images of p75NTR expression from normal and fibrotic liver tissue to visualize HSCs, showing elevated p75NTR expression (brown stain) of HSCs after bile duct ligation (B). Representative immunohistochemical images of GFP expression of liver sections from normal (upper panel) and fibrotic mice (lower panel) infected with the wild-type Ad.GFP (wt) as well as different modifications of the virus: binding of bispecific adapter molecule S11 alone (S11); S11 coupled with NGF peptide (S11-NGF_p_) and PEGylated Ad.GFP coupled with NGF peptide (PEG-NGF_p_). Uninfected mice livers served as control (control). Images (40x) display GFP expression of Ad infected cells (arrows). (C) Semiquantitative RT-PCR analysis of GFP expression in brains and lungs from representative normal and fibrotic (BDL) mice. GAPDH was used as positive control. Tissues from uninfected mice served as negative control. (D) Plasma levels of liver enzymes (AST and ALT) are as indicated. (E) NGF_p_ does not induce p75 receptor activation. Phase contrast images of PC12 cells treated with mature neural growth factor (NGF, 50 ng/ml) or NGF_p_ (alone or conjugated, 50 ng/ml). In contrast to mature NGF (positive control) none of the NGF_p_-treated cells shows neurite outgrowth. Magnification 20×.

To verify the *in vivo* observations, we further investigated the expression and cellular localization of GFP in infected livers by immunohistochemistry. Analysis of p75NTR expression revealed an increased expression of p75NTR in HSCs of fibrotic livers compared to normal liver tissue (Fig. 6A). In accordance with the *in vivo* fluorescence microscopic observations, wild-type virus efficiently infected hepatocytes of normal livers as shown in Fig. 6B by numerous GFP positive hepatocytes. In contrast, transduction rate in injured livers was less, concurrently infecting more nonparenchymal cells than hepatocytes (Fig. 6B).

In addition, an immense reduction of gene delivery as assessed by low GFP positivity was observed in hepatocytes of mice injected with S11-modified Ad.GFP. However, by targeting the virus with S11-NGF_p_, GFP expression was induced moderately in hepatocytes and enhanced in nonparenchymal liver cells, which is most prominent in liver tissue of BDL-mice. These GFP positive nonparenchymal cells were located along the sinusoids near the portal triads or in midzonal areas of the lobules. The selective infected cells were always matching with the morphology and localization of p75NTR-expressing HSCs.

PEGylation of the adenoviral vector resulted in non-hepatocellular transduction (data not shown). After exposure of the mice to NGF_p_-PEGylated viral vector, HSC transduction rate appeared to be moderately higher in BDL-mice compared to normal liver. Apart from their localization in the liver, intravenously injected viruses were not found in other tissues such lungs and brain (Fig. 6C). As demonstrated by the detection of ALT and AST levels in the plasma of treated normal and BDL-mice, no liver toxicity was observed for any of the utilized viruses.

Furthermore, we assessed whether addition of NGF_p_ itself has any impact on the activation and survival of p75NTR-positive cells using PC12 cells as model system for NGF-induced neuronal differentiation when cultured in serum free media. As shown in Fig. 6E, neurite outgrowth was evident only in the presence of mature NGF but not after treatment with NGF_p_ alone (NGF_p_, S11-NGF_p_) or conjugated (Ad.GFP_S11-NGF_p_). These data suggest that there are no p75NTR-mediated effects on HSC function through the NGF_p_ peptide used.

In summary, coupling of NGF_p_ to Ad via S11 and/or PEGylation resulted in a reduced hepatocellular tropism and an enhanced adenoviral-mediated gene transfer to HSCs. Transduction efficiency of both specific Ads was uniformly better in fibrotic livers, whereas Ad.GFP-S11-NGF_p_ transduced activated HSCs with higher frequency.

## Discussion

The two-step entry pathway of Ad vectors is initiated by the particle's fiber binding to cellular CAR and followed by the interaction of the RGD motif in the penton base with αβν3/5 integrin triggered internalization [14]. The viral liver uptake is the most important barrier for systemic delivery of Ad vectors. The use of adenovirus for anti-fibrotic liver gene therapy [34] requires targeting specifically to HSCs, without virus dissemination and an extended plasma circulation time [35]. In this approach, we compared two commonly applied *in vivo* targeting strategies showing for the first time a selective adenoviral transfection of HSCs in normal as well as in fibrotic livers.

p75NTR is a death domain-bearing member of the TNF receptor family and a receptor for the neurotrophin peptide family of which nerve growth factor (NGF) is the paradigm member [36]. Beside its expression in neuronal tissues [37], [38], previous studies have shown that components of the neurotrophin axis, including p75NTR, are expressed in non-neuronal tissues [39]–[41] including the liver [2]–[4]. Quiescent HSCs express low levels of p75NTR, whereas hepatocytes do not express this receptor [4]. Interestingly, the expression of p75NTR is rapidly increased after the experimental outset of hepatic fibrosis in rodents as well as in *in vitro* cultured HSCs [2]. The induction of p75NTR in activated HSCs during fibrogenesis prompted us to examine the relevant binding sequence of the NGF ligand to p75NTR as a targeting moiety [28], obtaining via a computational sequence alignment the NGF peptide for HSC targeting. To date, only few methods have been developed for HSC targeting which could lead to successful aiming at those and minimizing the toxicity of anti-fibrotic agents being capable to be used as novel medications [5]–[8], [42], [43]. These mostly *in vitro* results are promising, but additional studies need to be performed to achieve optimal targeting of HSCs in animal models.

In the present study, we used two different methods to construct selective infectious virus particles by coupling NGF_p_ to adenoviral structures. We verified the binding efficiency and tested the cell specificity of these constructs *in vitro* in primary cultured liver cells as well as *in vivo* by intravital fluorescence microscopy of the liver.

**Figure 7:**
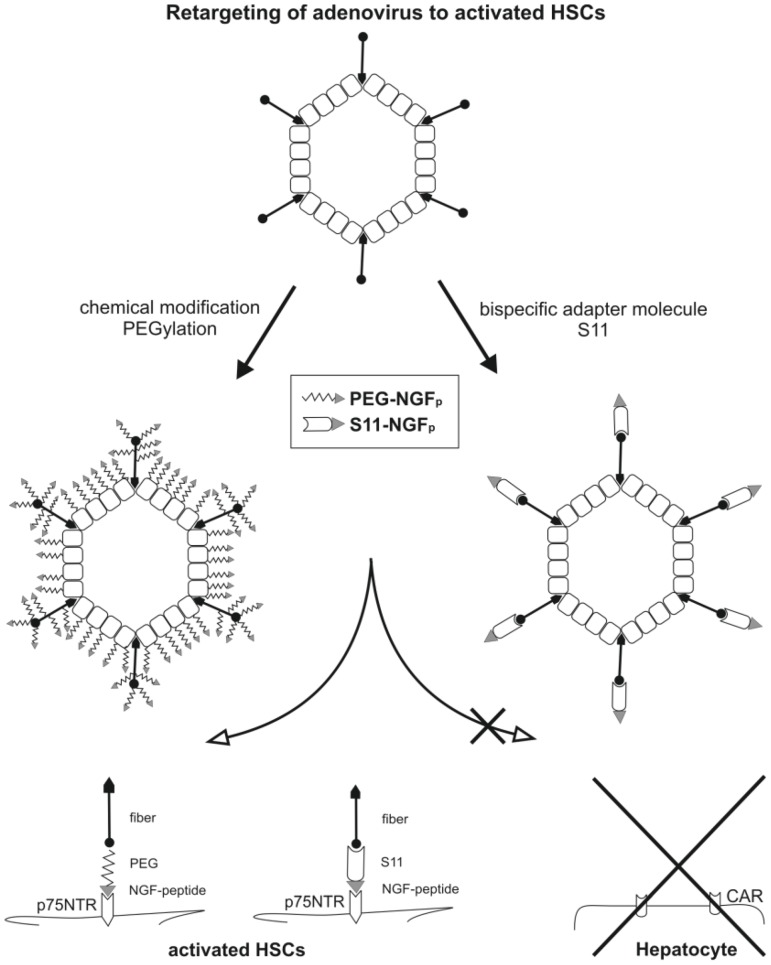
Two approaches to specifically target HSCs. Illustration of different HSC-specific retargeting strategies for adenoviral vectors by linking NGF_p_, specific for p75NTR, to viral surface proteins via chemical coupling (PEGylation) or adapter molecules (S11). The two different methods lead to masking of distinct viral structures. The “cloud” formed by PEGylation shields the whole virus surface including CAR- and RGD-binding motifs, while coupling of the S11 adapter molecule only screens the virus fiber knob necessary for interaction with CAR.

In the first strategy, we conjugated the adenoviral capsid with bifunctional PEG via NGF_p_ (Fig. 7). Although the general concept of the modification of Ad with bifunctional PEG and homing device was postulated earlier [29], [44], Ogawara and colleagues showed for the first time that this retargeting strategy leads to selective *in vivo* gene transfer [45]. On the other hand, PEGylation prolongs the blood circulation time [46], [47], thereby increasing the efficiency of Ad gene delivery to specific liver cells [48], [49]. In the second strategy, an antibody conjugate was developed whereby a single-chain antibody fragment (S11) binds to Ad fiber knob and its cross-linking to NGF_p_ targets the HSC-specific p75NTR (Fig. 7). This coating blocks the interaction with CAR and thus ablates its native tropism [50], [51]. The S11 fused to a cell receptor specific peptide was already described by Schoemaker and colleagues [7] who developed a strategy to target activated HSCs via a PDGF-β-receptor-specific peptide. Gene transfer by Ad vectors bound to this adapter molecule reduced hepatocyte infection *in vitro*. Also, using this method, Haisma et al. showed an efficient infection of subcutaneous carcinoma and liver detargeting after systemic virus injection [25].

Healthy controls as well as mice with a common bile duct ligation (BDL) were used to specifically target HSCs by systemic Ad virus administration. By coupling NGF_p_ via the PEG or the S11 strategy, hepatocyte tropism of the viral particles was successfully altered in both mice groups as determined by colocalization of GFP expression with vitamin A autofluorescence in HSCs. Limited transduction efficiency in control mice was related to the low p75NTR expression on quiescent HSCs [2], [4]. In contrast, fibrotic livers infected with both specific viruses revealed a high number of GFP positive cells which were identified as HSCs. It is likely that the higher transduction efficiency in fibrotic livers was related to increased p75NTR expression on the surface of activated HSCs. Regarding the targeting efficiency, quantification of the *in vivo* analysis revealed that the S11-NGF_p_ coupled virus showed higher HSC driven transgene expression than the PEGylation strategy. This observation is possible due to the different methods of virus modification used (Fig. 7). While PEGylation forms a “cloud” around the vector shielding the particle from *in vivo* binding receptors and factors, the S11 adapter molecule only blocks the CAR binding to the fiber knob, leaving the integrin binding RGD motif accessible. This is compatible with the notion that co-receptors for virus uptake, such as integrins are consistent expressed in HSCs. However, a solely integrin-mediated virus uptake [17], [52], [53] in HSCs was not observed (Ad.GFP-S11). Furthermore, compared to chemical conjugation, the S11-NGF_p_ strategy offers additional technical advantages, including simplified production in eukaryotic cell systems as well as fast protein purification and subsequent vector modification. In addition, the adapter molecule strategy allows the application of different fusion proteins suitable to retarget Ads to other receptors by simply substituting the ligand, thereby offering a standardized method. The production of PEGylated virus, however, is more tedious and costly compared to the application of readily available adapter molecules. Taking together, these facts provoke us to focus on the S11 adapter molecule strategy for further therapeutic applications.

Previous studies have shown that p75NTR is widely expressed throughout the body, and that its expression can be upregulated by injury or disease, including liver cirrhosis [2], [3], [54]. Due to this ubiquitous expression, we additionally analyzed the distribution of intravenously administered modified Ads by GFP-mRNA expression in lung and brain. In fact, no detectable GFP expression was observed, which indicated that the delivery of modified Ads was HSC-specific. Moreover, neurite outgrowth assays suggested that our peptide targeting strategy does not influence HSC function.

Here we demonstrated that adenoviral-mediated targeting of HSCs via p75NTR, concurrently avoiding its binding to hepatocytes, might provide a potentially feasible and effective mechanism for therapeutic gene delivery to activated HSCs.
