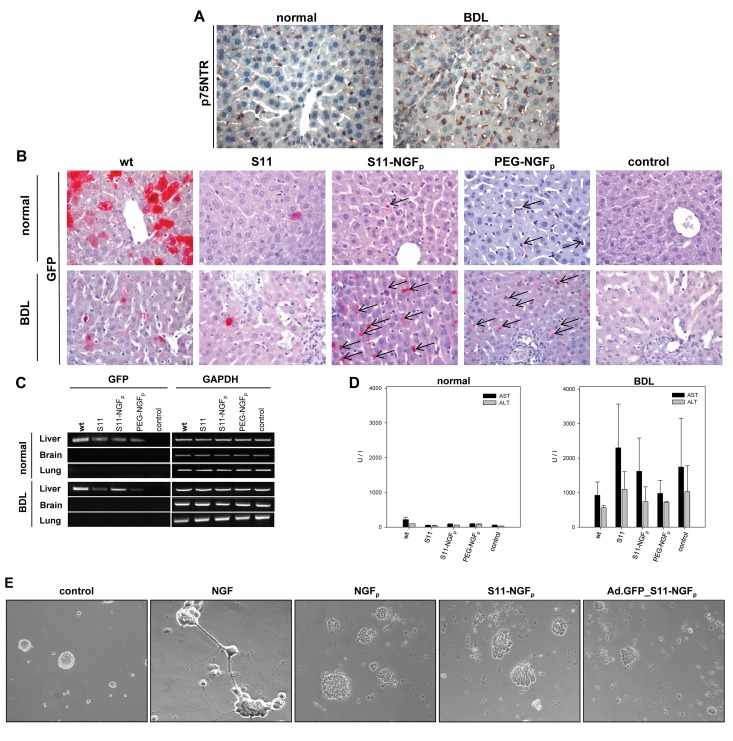# Correction: Development of Adenoviral Delivery Systems to Target Hepatic Stellate Cells In Vivo

**DOI:** 10.1371/annotation/981389e9-e5a1-4be6-bf55-841a7102a623

**Published:** 2013-07-31

**Authors:** Julia Reetz, Berit Genz, Claudia Meier, Bhavani S. Kowtharapu, Franziska Timm, Brigitte Vollmar, Ottmar Herchenröder, Kerstin Abshagen, Brigitte M. Pützer

There are panels missing from Figures 2, 5, and 6. Please see the correct versions of these figures at the following links:

Figure 2- 

**Figure:**
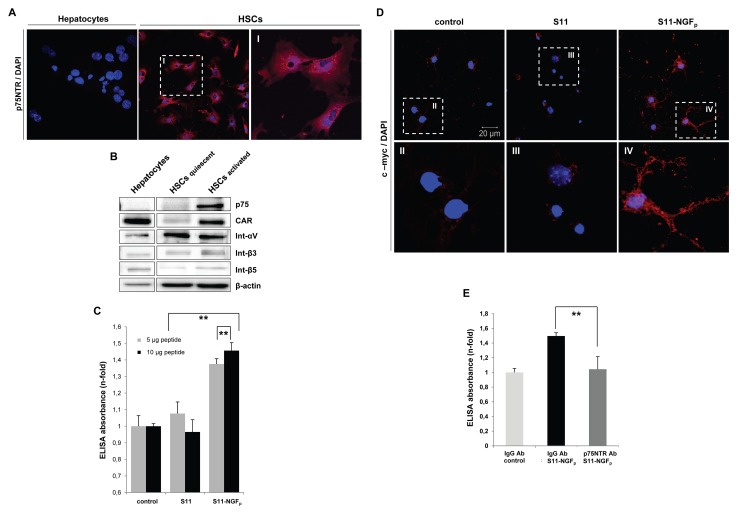


Figure 5- 

**Figure:**
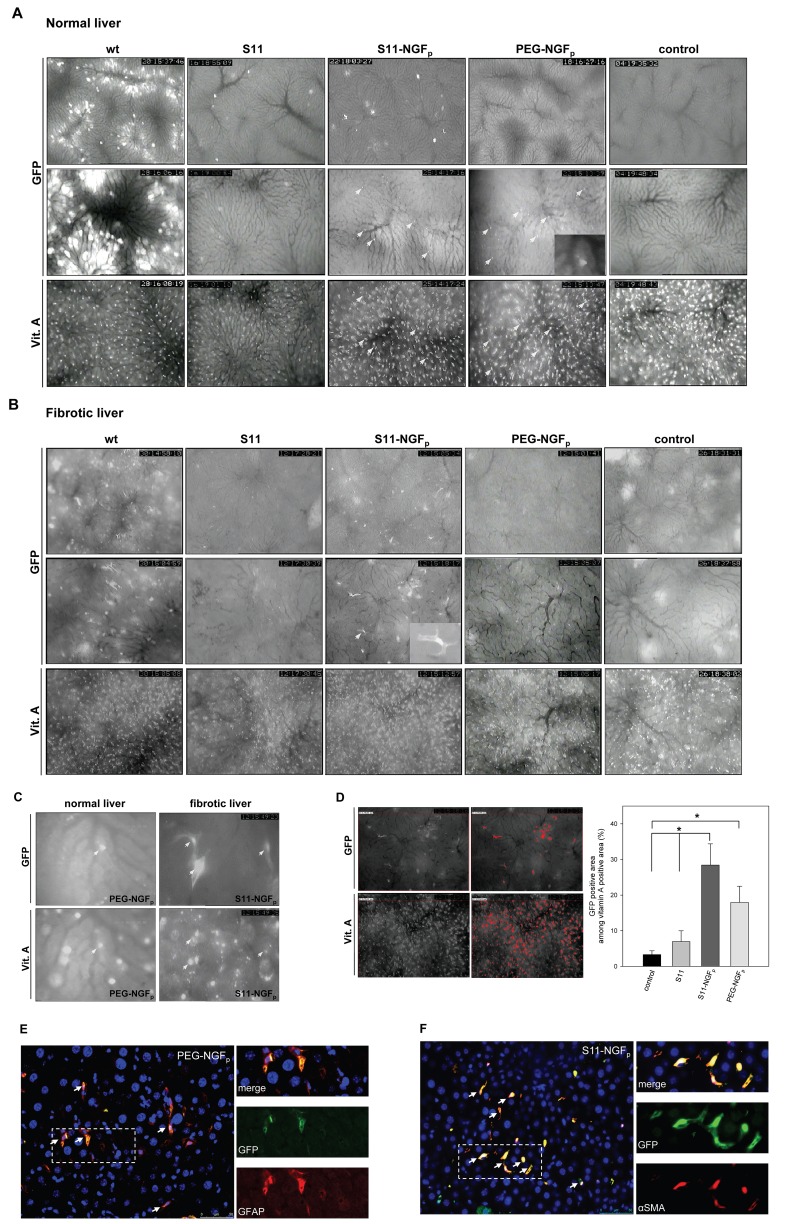


Figure 6- 

**Figure:**